# Administration of mesenchymal stromal cells before renal ischemia/reperfusion attenuates kidney injury and may modulate renal lipid metabolism in rats

**DOI:** 10.1038/s41598-017-08726-z

**Published:** 2017-08-17

**Authors:** Pauline Erpicum, Pascal Rowart, Laurence Poma, Jean-Marie Krzesinski, Olivier Detry, François Jouret

**Affiliations:** 1 Groupe Interdisciplinaire de Génoprotéomique Appliquée (GIGA), Cardiovascular Sciences, University of Liège, Liège, Belgium; 2 Division of Nephrology, University of Liège Hospital (ULg CHU), Liège, Belgium; 3 Department of Abdominal Surgery and Transplantation, University of Liège Hospital (ULg CHU), Liège, Belgium; 4 Groupe Interdisciplinaire de Génoprotéomique Appliquée (GIGA), CREDEC Unit, University of Liège, Liège, Belgium

## Abstract

Mesenchymal stromal cells (MSC) have been demonstrated to attenuate renal ischemia/reperfusion (I/R) damage in rodent models. The mechanisms of such nephro-protection remain largely unknown. Furthermore, the optimal timing of MSC administration has been poorly investigated. Here, we compare the impact of MSC injection 7 days before (MSCD − 7) *versus* 1 day after (MSCD + 1) renal I/R in rats. Control groups received equivalent volumes of saline at similar time-points (SD − 7 and SD + 1). Right nephrectomy was performed, and left renal ischemia lasted 45 min. After 48-hour reperfusion, we observed significantly improved renal function parameters, reduced apoptotic index and neutrophil/macrophage infiltration in kidney parenchyma, and lower expression of tubular damage markers and pro-inflammatory cytokines in MSCD − 7 in comparison to MSCD + 1 and saline control groups. Next, comparative high-throughput RNA sequencing of MSCD − 7 *vs*. SD − 7 non-ischemic right kidneys highlighted significant down-regulation of fatty acid biosynthesis and up-regulation of PPAR-α pathway. Such a preferential regulation towards lipid catabolism was associated with decreased levels of lipid peroxidation products, i.e. malondialdehyde and 4-hydroxy-2-nonenal, in MSCD − 7 *versus* SD − 7 ischemic kidneys. Our findings suggest that MSC pretreatment may exert protective effects against renal I/R by modulating lipid metabolism in rats.

## Introduction

Acute kidney injury (AKI) is a life-threatening clinical condition commonly observed in hospitalized patients, particularly in operative settings. Ischemia-reperfusion (I/R) injury represents one of the leading causes of AKI, and is induced by the transient interruption of renal blood flow. The abrupt drop in oxygen partial pressure and nutrient delivery leads to a cascade of cellular and tissular events, resulting in cytoskeleton disorganization, loss of cell polarity and dysfunction of membrane ion transporters. Subsequent reperfusion causes a massive production of reactive oxygen species (ROS), which are responsible for detrimental oxidation of proteins, lipids and nucleic acid in both epithelial and endothelial cells. Inflammation implying both innate and immune systems also contributes to the injury^1, 2^. Treatment of AKI currently relies on supportive manoeuvers^3^. Still, recent advances in the pathophysiology of renal I/R highlighted putative novel therapies, including cell-based therapy^4, 5^.

Mesenchymal stromal cells (MSC) represent a heterogeneous population of fibroblast-like adult multipotent cells which can be isolated from various sources, including bone marrow, umbilical cord, muscles and adipose tissue^6^. Their definition has been standardized: (i) adherence to plastic surfaces; (ii) ability to differentiate into adipocytes, chondrocytes and osteoblasts *in vitro*; (iii) combined expression of CD29 and CD90, CD73 and CD105 surface molecules and lack of expression of the hematopoietic markers CD45, CD34, CD14, CD79a, CD11b and HLA-DR^7–9^. Anti–inflammatory and immune-regulatory properties of MSC have been reported in numerous *in vitro* and *in vivo* studies^5, 10–12^. Moreover, MSC exert tissue repair function in damaged organ by reducing inflammation and stimulating vascular supply^4^. Their beneficial effect predominantly involves paracrine and endocrine pathways rather than trandifferentiation^13^. MSC-derived microvesicles may also allow horizontal transfers of mRNA, microRNA and proteins to their neighboring cells^14, 15^.

A number of experimental studies have provided promising data using MSC therapy in various models of I/R-related AKI^4^, and clinical trials are ongoing^16, 17^. Hence, MSC administration either immediately or 24 h after renal ischemia significantly improved renal function with higher proliferative and lower apoptotic indexes in anesthetized rats exposed to I/R injury^13^. In strong contrast, Perico N. *et al*.^18, 19^ reported on an engraftment syndrome characterized by AKI following MSC infusion in both rodent models and human clinical trials of kidney transplantation (KTx). KTx necessarily conveys renal I/R. These authors demonstrated that pre-transplant administration of MSC 7 days before KTx reduces neutrophil infiltration into kidney parenchyma and prolongs allograft survival in mice^19^. Outcome differences in pre- *versus* post-transplant administration of MSC may be explained by the differential homing location of MSC into the spleen and lymphoid organs *versus* the ischemic organ, respectively. Similarly, Merino A. *et al*. demonstrated, in a rat renal allograft model, that the optimal time schedule for MSC infusion to prevent acute rejection was 7 days before KTx^20^. MSC could thus exert contrasting effects depending on the timing of administration related to the injury, which, in turn, conditions the microenvironment^21, 22^.

In the field I/R-related AKI, the administration of MSC prior to the ischemic insult may mimic ischemic preconditioning (IPC)^23^. IPC is thought to exert its nephro-protection in 2 consecutive, immediate and delayed, phases^24^. The delayed phase of IPC initiates a complex genomic and proteomic response, and is considered as the most efficient one in terms of nephro-protection^24^. The present study aims at (i) determining the impact of the timing of MSC infusion, i.e. before *versus* after I/R, on structural and functional parameters of kidney injury in rats, and (ii) identifying the cellular pathways implicated in MSC-induced IPC, including their impact on kidney metabolism.

## Results

### In comparison to saline infusion, MSC administration 7 days prior to renal I/R helps preserve renal function, whereas MSC administration 1 day after I/R worsens renal function

**Figure 1:**
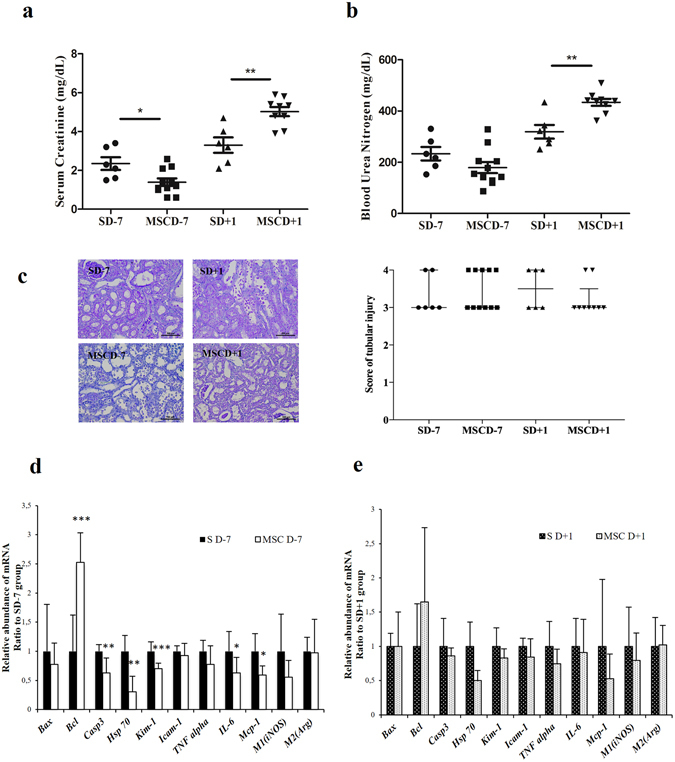
Kidney functional and structural parameters and markers of apoptosis and cell proliferation in renal parenchyma after ischemia/reperfusion according to the timing administration of MSC. (**a**–**d**) *Lewis* rats underwent i.v. injection of MSC 7 days before (MSCD − 7, n = 11) or 1 day after (MSCD + 1, n = 9) renal I/R. Control group received equal volume of saline at the same time-points (SD − 7, n = 6; SD + 1, n = 6) (**a**,**b**) Serum creatinine (SCr) and blood urea nitrogen (BUN) levels were measured at 48 h *post* renal I/R. (**c**) Histologic damage was graded on PAS-stained kidney sections following Jablonski score^63^. Results are shown as medians and interquartile range. (**d**,**e**) Real-time qPCR quantification of mRNA expression levels of *Bax*, *Bcl-2*, *Caspase-3* (*Casp3*), *Heat-Shock Protein* 70 (*Hsp70*), *Kidney Injury Molecule* 1 (*Kim-1*), *Intercellular Adhesion Molecule 1* (*Icam-1*), *Tumor Necrosis Factor alpha* (*Tnfα*), *Interleukine* 6 (*IL-6*), *Monocyte Chemotactic Protein 1* (*Mcp-1*), *inducible NO synthase* (*iNOS*) *and arginase* (*Arg*) in the kidney after 45 minutes of ischemia followed by 48 hours of reperfusion in SD − 7 versus MSCD − 7 groups (**d**) and in SD + 1 versus MSCD + 1 groups (**e**). The mRNA expression levels were standardized using *Gapdh* as housekeeping gene. Significant differences are indicated, *p ≤ 0.05, **p ≤ 0.01 and ***p ≤ 0.001.

*Lewis* rats were categorized in 4 groups. Group 1 (MSCD − 7, n = 11) and group 3 (MSCD + 1, n = 9) received caudal i.v. injection (tail vein) of MSC (1.5 × 10^6^ in 1 mL saline) 7 days before or 1 day after renal I/R, respectively. Control group 2 (SD − 7, n = 6) and group 4 (SD + 1, n = 6) received equal volume of saline at similar time-points. Right nephrectomy and left renal 45-min ischemia (by clamping the renal pedicle) were simultaneously performed. Blood samples were collected from inferior *vena cava* at 48 hours post-reperfusion. Following such a protocol of renal I/R, one-way analysis of variance (ANOVA) demonstrated statistically significant differences in serum creatinine (SCr; *p* ≤ 0.001) and blood urea nitrogen (BUN; *p* ≤ 0.001) levels among the 4 groups. Mean SCr reached 1.39 ± 0.69 mg/dL in MSCD − 7 group *versus* 2.35 ± 0.80 mg/dL in SD − 7 group (*p* ≤ *0*.*05*) (Fig. 1a). Mean BUN levels in MSCD − 7 and SD − 7 groups were 179.53 ± 75.24 mg/dL and 233.40 ± 64.56 mg/dL, respectively (*p* = 0.155) (Fig. 1b). No statistically significant differences between MSCD − 7 and SD − 7 groups were found using Jablonski’s histological score for acute tubular necrosis (Fig. 1c). In MSCD + 1 group, SCr and BUN levels were significantly higher than in SD + 1 group, with respective mean SCr values of 4.85 ± 0.70 mg/dL and 3.27 ± 0.97 mg/dL (*p* ≤ *0*.*01*) and respective mean BUN values of 441.65 ± 46.40 mg/dL and 319.22 ± 65.46 mg/dL (*p* ≤ *0*.*01*) (Fig. 1a,b). No difference in Jablonski’s severity score was observed between MSCD + 1 and SD + 1 groups (Fig. 1c). Comparative analyses between MSCD − 7 and MSCD + 1 are shown in Supplementary Figure 1.

### In comparison to saline infusion, MSC administration 7 days before renal I/R reduces neutrophil and macrophage infiltration, apoptosis and cell proliferation, while MSC administration 1 day after I/R increases apoptosis and cell proliferation

**Figure 2:**
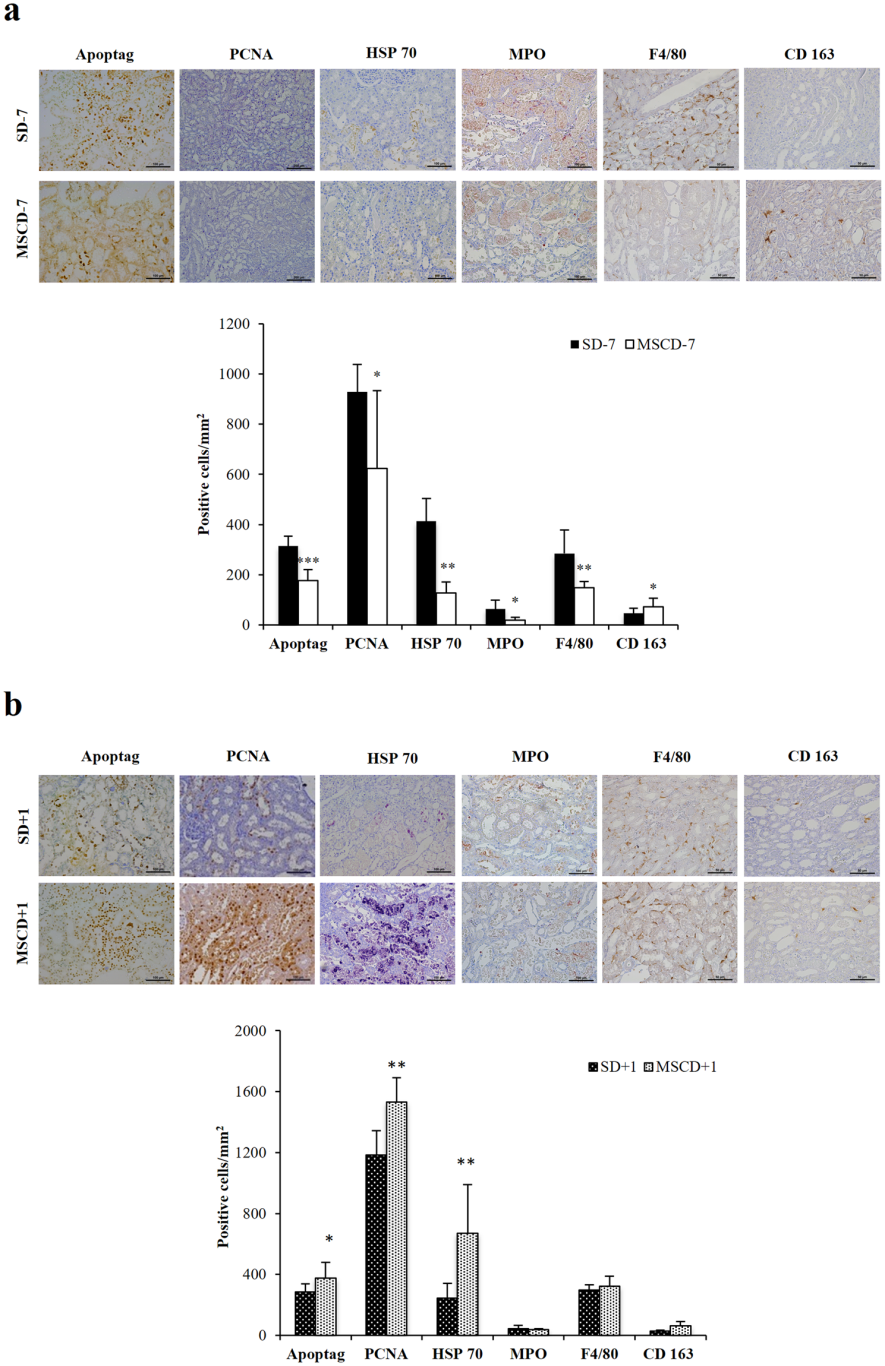
Quantification of markers of apoptosis, cell proliferation, inflammation in renal parenchyma after ischemia/reperfusion according to the timing administration of MSC. Immunohistochemistry and quantifications for Apoptag, proliferative cell nuclear antigen (PCNA), Heat-Shock Protein 70 (HSP70), myeloperoxidase (MPO), F4/80 and CD163 in SD − 7 and MSCD − 7 ischemic kidneys (**a**) and in SD + 1 and MSCD + 1 ischemic kidneys (**b**). One-way analysis of variance was performed among groups. Significant differences are indicated, *p ≤ 0.05, **p ≤ 0.01 and ***p ≤ 0.001.

Following renal I/R, the quantification of tubular cells expressing proliferating cell nuclear antigen (PCNA) and heat-shock protein 70 kDa (HSP70) is classically used to assess the severity of acute tubular necrosis^25^. Apoptosis was measured using TUNEL assay. Here, the administration of MSC at D − 7 was associated with a significantly reduced number of HSP70-positive (*p* ≤ 0.01), PCNA-positive (*p* ≤ 0.05) and apoptotic cells (*p* ≤ 0.001) along renal tubules (Fig. 2a), as well as a lower number of myeloperoxidase (MPO)-positive polymorphonuclear neutrophils infiltrating kidney parenchyma (*p* ≤ 0.05), in comparison to the control SD − 7 group. The number of F4/80-positive macrophages was higher in SD − 7 *versus* MSCD − 7 ischemic kidneys. Conversely, CD163-positive M2 macrophages were more numerous in ischemic kidneys exposed to MSCD − 7 compared to saline exposure (SD − 7) (Fig. 2a). No significant difference in neutrophil and macrophage recruitment was found between MSC D + 1 and SD + 1 groups (Fig. 2b). By contrast, HSP70-expressing (*p* ≤ 0.01) and PCNA-immunoreactive (*p* ≤ 0.01) epithelial cells were more numerous in MSCD + 1 than in SD + 1 kidneys. The number of TUNEL-positive cells was significantly increased in MSCD + 1 than SD + 1 groups (*p* ≤ 0.05) (Fig. 2b).

### In comparison to saline infusion, MSC administration 7 days before renal I/R is associated with a significantly decreased expression of pro-apoptotic factors and pro-inflammatory cytokines at the mRNA level

The mRNA expression levels of anti-apoptotic (*Bcl-2*) and pro-apoptotic (*Bax* and *Casp3*) markers, I/R severity scorers (*Hsp7*0 and *Kim1*) and pro-inflammatory cytokines (*Mcp-1*, *Icam-1*, *Il-6* and *Tnfα*) were comparatively quantified using real-time RT-qPCR. In comparison to SD − 7 controls, MSC administration at D − 7 was associated with a significant reduction of renal mRNA expression of *Casp3* (*p* ≤ *0*.01), *Hsp70* (*p* ≤ *0*.*01*), *Kim-1* (*p* ≤ 0.001), *Il-6* (*p* ≤ 0.05), *Mcp-1* (*p* ≤ 0.05) as well as a significant increase of *Bcl-2* mRNA expression (*p* ≤ 0.001) (Fig. 1d). In D + 1 groups, no difference was found in mRNA expression levels of *Bax*, *Bcl-2*, *Casp3*, *Hsp7*0, *Kim-1*, *Icam-1*, *Tnf-α* and *Il-6* between MSCD + 1 and SD + 1 ischemic kidneys (Fig. 1e).

### Transcriptomics indicate a down-regulation of fatty acid biosynthetic pathways at day 7 post-administration of MSC

**Figure 3:**
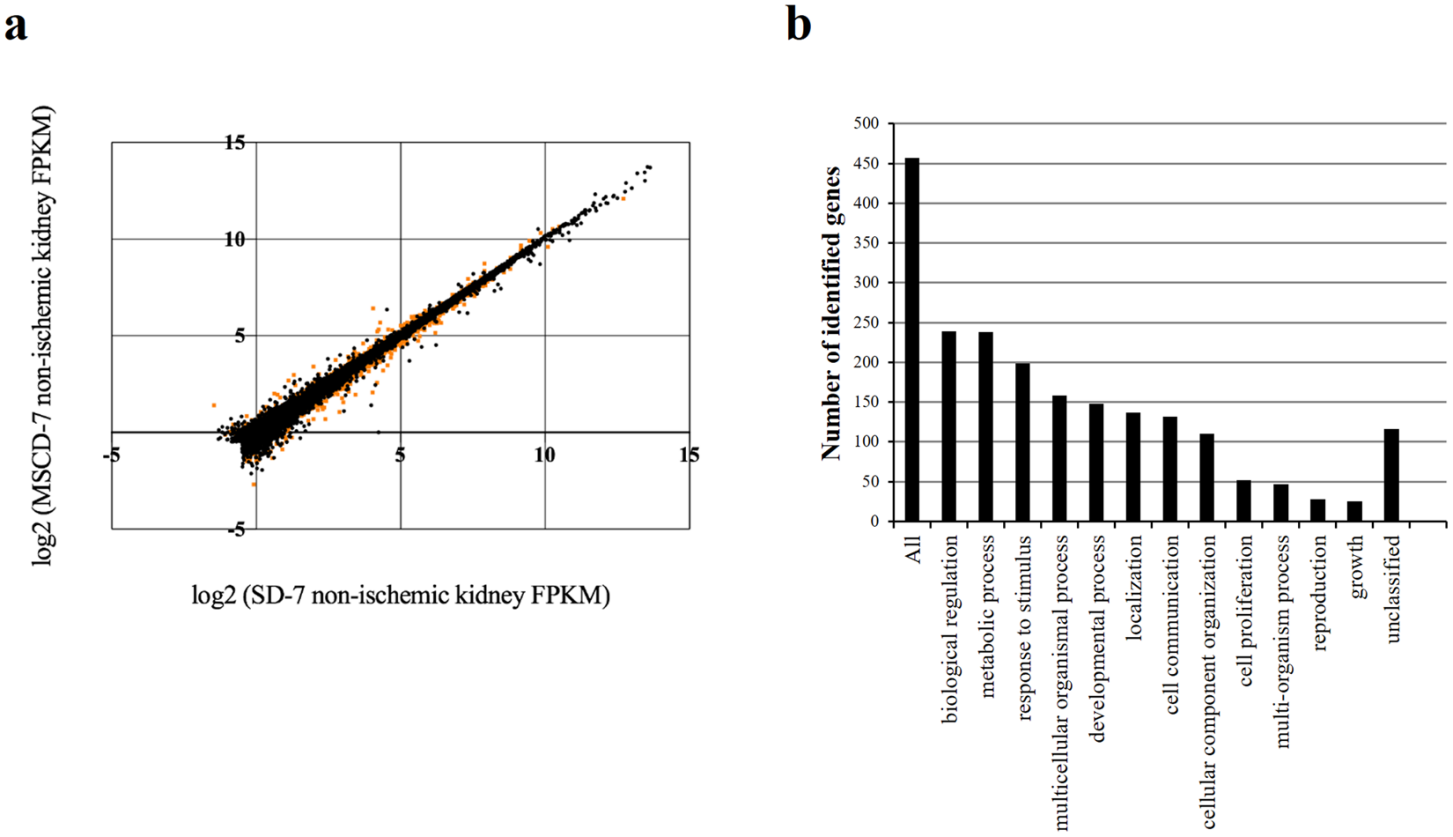
Illumina high-throughput RNA sequencing and Gene Ontology slim classification analyses. (**a**) Differential gene expression analysis obtained with BaseSpace^®^ Cufflinks Assembly. Scatter plot of the log2 (Fragments per Kilobase of sequence Per Million mapped reads, FKPM) counts of genes for control (SD − 7) and MSC-treated (MSCD − 7) non-ischemic kidneys. Dots represent the 25908 genes differentially assessed, with orange dots corresponding to the significantly differentially expressed genes (False Discovery Rate (FDR), <0,05). (**b**) Gene Ontology classification for biological processes of the significantly differentially expressed genes successfully mapped to a gene symbol (WEB-based GEne SeT AnaLysis Toolkit).

**Figure 4:**
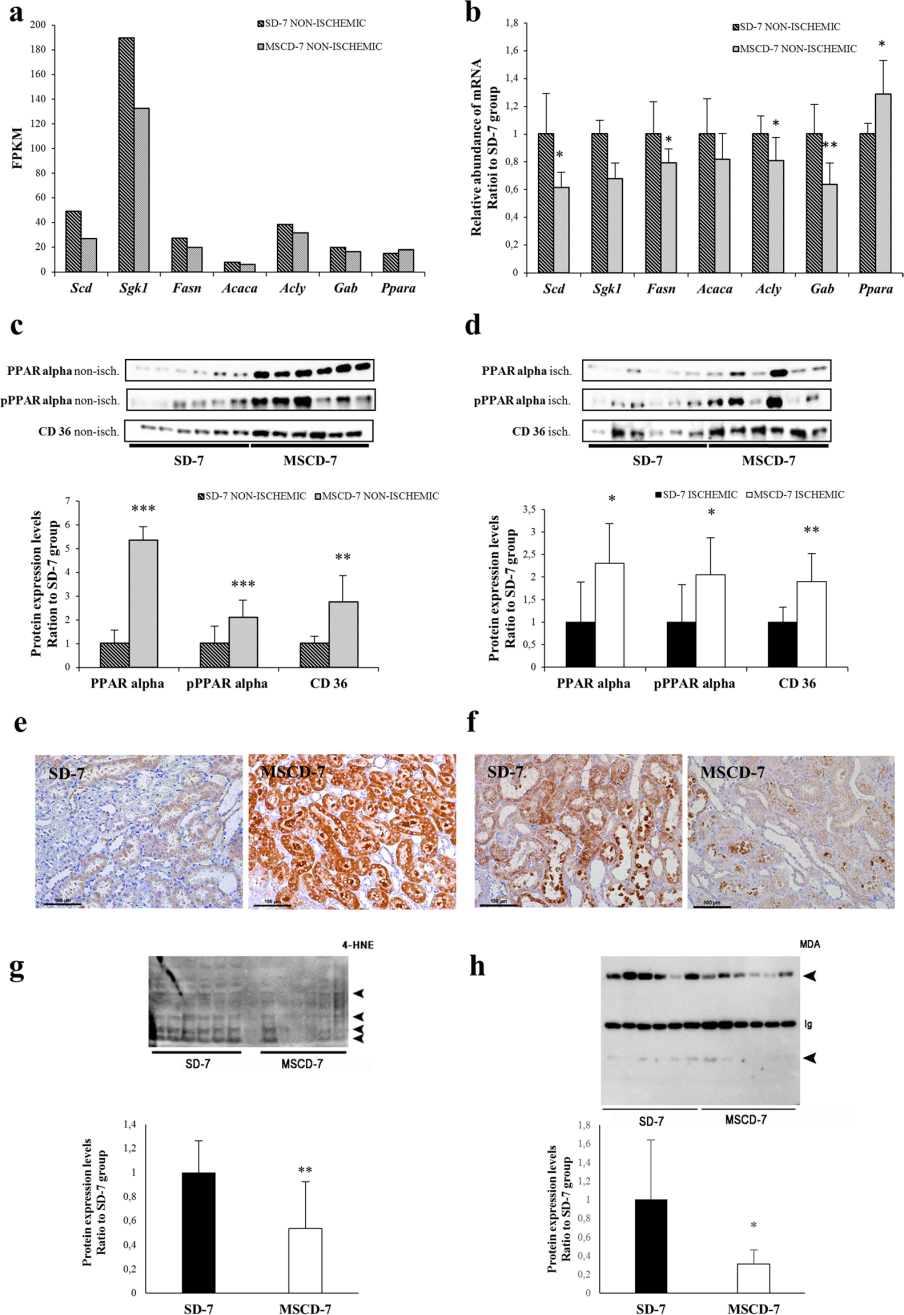
Impact of MSC on lipid metabolism. (**a**) Significantly differentially expressed genes involved in fatty acid biosynthesis and nuclear receptor in lipid metabolism pathways in non-ischemic kidneys exposed to MSC (MSCD − 7, n = 6) *versus* saline (SD − 7, n = 6) 7 days before renal I/R, on the basis of the high-throughput RNA-sequencing. Data are shown in Fragments Per KiloBase per Million of mapped reads value. (**b**) RT-qPCR analysis of the genes corresponding to panel (a). The mRNA expression levels were standardized using GAPDH as housekeeping gene. (**c**,**d**) Quantification of PPARα, phospho-PPARα and CD36 expression in non-ischemic (panel c) and ischemic (panel d) kidneys. (**e**) Immunohistochemistry for FAT/CD36 in non-ischemic kidneys. (**f**–**h**) Representative immunochemistry of malondialdehyde (MDA) in renal parenchyma and quantitative immunoblotting of 4-hydroxy-2-nonenal (4-HNE) and MDA modified proteins (arrowheads) in ischemic kidneys of SD − 7 and MSCD − 7 groups (*Ig*, *Immunoglobulins*). Data are presented as mean ± standard deviation. Significant differences are indicated, *p ≤ 0.05, **p ≤ 0.01 and ***p ≤ 0.001 *versus* appropriate control group.

High-throughput RNA sequencing technology was used to probe the differential transcriptomic renal profiles of rats infused with MSC compared to saline (Fig. 3a,b). From the methodological point of view, messenger RNAs were extracted from the right kidneys of rats exposed (MSCD − 7, n = 6) or not-exposed (SD − 7, n = 6) to MSC 7 days before sampling. These kidneys were not exposed to ischemia, and were harvested before the 45-min ischemia of the contralateral kidneys. After (i) mapping the reads onto the rat genome (rn5), (ii) transcriptome reconstruction and (iii) abundance calculation, differentially expressed gene were identified using Cufflinks. A total of 25908 genes were assessed for differential expression calculation between MSCD − 7 and SD − 7 non-ischemic groups. Among these genes, 748 genes were found to be significantly differentially expressed (False Discovery Rate, FDR < 0.05): 493 and 255 genes were down- and up-regulated in MSCD − 7 group, respectively (Fig. 3a). To allow the identification of relevant groups of related genes sharing biological functions or pathways, functional enrichment analysis was performed using Database for Annotation, Visualization and Integrated Discovery (DAVID) and WEB-based GEne SeT AnaLysis Toolkit^26, 27^. Using gene ontology analysis via WebGestalt^28^, 457 identified genes with unambiguous gene symbol were allocated to ontology categories depending on their biological functions (Fig. 3b). Using Overrepresentation Enrichment analysis based on Wikipathway Enrichment Categories, we found that the metabolic pathways mostly affected by MSC pre-infusion are implicated in adipogenesis, insulin signaling, fatty acid (FA) biosynthesis, IL-6 signaling, B-cell receptor signaling, IL-3 pathway, proteasome degradation, and nuclear receptors involved in lipid metabolism (Table 1). Because of previous reports suggesting renal lipotoxicity as a key mechanism in I/R AKI^29^, we selected 6 downregulated genes (Stearoyl-CoA desaturase (*Scd*), Serum- and glucocorticoid-inducible kinase 1 (*Sgk1*), Fatty acid synthase (*Fasn*), Acetyl-CoA carboxylase (*Acaca*), ATP citrate lyase (*Acly*), GRB2-associated binding protein (*Gab*)) and 1 upregulated gene (Peroxisome proliferator-activated receptor alpha (*Ppara*)) to validate the RNA-Sequencing differential data between MSCD − 7 *versus* SD − 7 groups using real-time (RT)-qPCR. The mRNA expression level of *Ppara* was significantly increased in MSCD − 7 group compared to SD − 7 group, whereas the mRNA expression levels of *Acly*, *Gab*, *Fasn*, *and Scd* were significantly decreased in MSCD − 7 group, as suggested by the high-throughput RNA sequencing (Fig. 4a,b).

**Table 1:** Metabolic pathways involved in MSC-mediated conditioning based on the high-throughput RNA sequencing (WEB-based Gene Set AnaLysis Toolkit).

Pathway name	Number of genes in the category	Number of reference genes in the category	Adjusted p-value
Downregulated pathways			
Adipogenesis	10	129	5 × 10^−7^
Insulin signaling	9	158	1.36^−5^
Fatty acid biosynthesis	5	28	1.36^−5^
IL-6 signaling pathway	7	114	0.0001
B cell receptor signaling pathway	8	199	0.0003
ErbB signaling pathway	5	60	0.0003
IL-3 signaling pathway	6	110	0.0004
EGFR1 signaling pathway	8	213	0.0004
MAPK signaling pathway	7	165	0.0004
Upregulated pathways			
Nuclear receptors in lipid metabolism and toxicity	4	39	0.0001
Proteasome degradation	2	59	0.0480
Translation Factors	2	47	0.035

### In comparison to saline infusion, MSC administration induces the expression and activation of PPARα and FAT/CD36, and attenuates I/R-associated lipid peroxidation

Using immunoblotting, we found that PPARα and phosphorylated PPARα were significantly increased in MSC-exposed (MSCD − 7) kidneys in comparison to saline-infused controls, in both non-ischemic (*p* ≤ *0*.*001*) and ischemic (*p* ≤ 0.05) conditions. Additionally, we observed an induced expression of FAT/CD36 in kidneys of MSC-*pre-*infused animals, significantly contrasting with controls (*p* ≤ 0.01) (Fig. 4c,d). Immunohistochemistry revealed a preferential localization of FAT/CD36 in the cytoplasm of the epithelial cells lining the proximal tubules (Fig. 4e). Finally, we found that MSC administration 7 days prior to I/R significantly attenuated lipid peroxidation in kidney parenchyma, as quantified by malondialdehyde (MDA) and 4-hydroxy-2-nonenal (4-HNE) modified proteins (Fig. 4f–h).

## Discussion

Cell-based therapy has emerged as a promising strategy for the treatment of various conditions, including AKI. Preclinical and pilot clinical studies support that MSC may attenuate AKI and accelerate recovery^4, 5^. Still, controversies remain concerning the modalities of MSC infusion^8, 30^. Here, we show that infusing rats with 1.5 × 10^6^ MSC 7 days before renal I/R improves renal function, decreases inflammation and apoptosis in kidney parenchyma, and may modulate FA biosynthesis. Conversely, injecting MSC after renal I/R is deleterious in our model, with worsened kidney function and higher scores of inflammation and apoptosis. These observations further emphasize the impact of the timing of MSC administration with respect to organ injury^10, 22^. Furthermore, exposure to MSC may participate to renal conditioning, with activation of the PPARα/CD36 pathway. Our model is limited to 48-hour reperfusion, and may not fully reflect the complexity of I/R-associated AKI observed in humans. Additionally, lipid metabolism is different between rats and humans, particularly concerning *de novo* lipogenesis^31^. Finally, MSC used in the present study were suspended in saline at the time of the i.v. delivery in order to avoid infusing rats with culture medium (enriched with multiple and various solutes and metabolites, including fetal bovine serum and antibiotics). Saline infusion at the time of renal I/R may alter renal perfusion because of its supraphysiological concentration of chloride leading to vasoconstriction of glomerular arterioles^32, 33^. This methodological particularity may partly explain the discrepancy between our observations and previous reports^4^.

The influence of MSC infusion timing has been poorly investigated, although *in vitro* and *in vivo* data have demonstrated that the environment strongly influences MSC phenotype and properties^22, 34^. Indeed, MSC-associated immunomodulation may be due to a stepwise activation induced by soluble factors not constitutively expressed by MSC but triggered by inflammation, including IFN-γ, TNF-α, IL1α and IL1β^22, 35^. The *in vitro* addition of MSC to CD4 lymphocytes exerts different consequences upon the status of target T-cells. Late addition of MSC after T-cell induction only suppresses Th1 cell lineage, subsequently expanding pro-inflammatory Th17 cells^36^. Casiraghi F. and colleagues demonstrated *in vivo* in a sensitized mouse model of KTx that *pre-*transplant administration of MSC prolonged allograft survival by promoting the expansion of donor-specific regulator T-lymphocytes, whereas *post-*transplant administration of MSC was associated with early graft dysfunction characterized by increased renal recruitment of neutrophils and *in situ* complement activation^19^. Similar observations of “engraftment syndrome” were made in pilot clinical trials including kidney transplant recipients^18, 37^. The authors hypothesized that KTx triggers graft inflammation, which in turn causes the recruitment of MSC to the transplant and favors MSC differentiation towards a pro-inflammatory phenotype.

The nephro-protection induced by MSC infusion before renal I/R may mimic “delayed remote conditioning”^24^. Indeed, MSC do not transdifferentiate into mature tissue, but rather act via the secretion of endocrine or paracrine factors^38^. Such humoral alert may activate signal transduction pathways implicated in cell resistance to I/R and in cell recovery. Interestingly, we observed a significant impact of MSC on FA biosynthesis and lipid peroxidation in kidney parenchyma. Lipotoxicity has been well documented in various models of renal I/R injury^39, 40^. Particularly, I/R injury-associated accumulation of triglycerides (TG) depends on (i) FA mobilization from membrane phospholipids by phospholipase A2 (PLA2)^40, 41^, (ii) inhibition of TG degradation^42^, and (iii) acceleration of TG synthesis from free FA^42^. Exposure of proximal tubule cells to the mitochondrial-blocking agent, antimycin A (as an *in vitro* model of ischemia), upregulates TG formation, namely via fatty acid synthase (FAS)^42^. In our model, *in vivo* administration of MSC caused a down-regulation of key enzymes in FA biosynthesis, including FAS, as suggested by high-throughput RNA sequencing and confirmed by RT-qPCR. Additionally, MSC infusion activates PPARα pathway. PPARα is a member of the nuclear receptor superfamily of ligand-activated transcription factors, regulating lipid homeostasis, inflammation and vascular integrity^43^. PPARα is highly expressed in metabolically active tissues, including renal proximal tubules^44^. In case of renal I/R, PPARα expression decreases^45^, along with the rapid inhibition of microsomal and peroxisomal FA oxidation^46^. Conversely, pharmacological stimulation of PPARα by fibrates has been shown to attenuate I/R-associated AKI and accelerate kidney recovery^45, 47–49^. In rats, administration of PPARα agonist 5 days prior to renal I/R beneficially modulates the genes involved in FA oxidation, thereby preserving kidney structure and function^50^. Likewise, MSC infusion 7 days prior to renal I/R appears to up-regulate PPARα and attenuates AKI severity. In line with our observations, MSC have been previously shown *in vitro* to prevent lipotoxicity and improve cell viability and regeneration in high palmitic conditions^51^. MSC-induced nephro-protection may thus be linked to reduced lipotoxicity and lipid peroxidation at the time of renal I/R injury, as supported by a significant reduction in the abundance of MDA and 4-HNE modified proteins observed in our model.

Besides modulating FA oxidation, PPARα also regulates transmembrane import of FA in a tissue-specific manner^52–54^. FAT/CD36 is a class B scavenger receptor broadly expressed, including in monocytes/macrophages and smooth muscle cells. This receptor has been implicated in several biological processes, and may respond to various ligands, such as thrombospondin-1, modified LDL and long-chain fatty acids. FAT/CD36 participates to the regulation of innate immunity, FA transport and angiogenesis^55^. Focusing on lipid metabolism, FAT/CD36 may be involved in mitochondrial FA oxidation, both at rest and in cases of metabolic challenges. In kidneys, FAT/CD36 is mostly expressed in proximal tubular cells and podocytes, where it could contribute to glomerulosclerosis and albuminuria in diabetic nephropathy^56^. Palmitic acid-driven upregulation and translocation of CD36 from the cytosol to the plasma membrane lead to an increase in lipid uptake, ROS production and apoptosis in podocytes of patients with diabetic nephropathy and hyperlipidemia. In the field of I/R-related injury, controversies remain concerning the role of FAT/CD36. In mouse, the loss of FAT/CD36 results in impaired FA oxidation and reduced levels of glycogen, triglycerides and ATP in the heart. Consequently, CD36-deficient hearts are more susceptible to I/R injury because of lower energy storages before I/R and defective energy regeneration after I/R^57^. In strong contrast, hyperlipidemia exacerbates I/R injury in brain by promoting CD36-mediated inflammation in ApoE knock-out mice under high-fat diet^58^. The role of PPARα in governing the expression of FAT/CD36 in kidney has been poorly investigated to the best of our knowledge. In our study, MSC infusion is associated with both the activation of PPARα and the upregulation FAT/CD36 in both non-ischemic and ischemic conditions in comparison to saline infusion.

In addition to renal lipid metabolism, signaling pathways involved in inflammation modulation are also influenced by MSC pre-infusion, as suggested by our observations. Both innate and adaptive immune responses crucially contribute to the pathophysiology of renal I/R^5^. Activation of Toll-like receptors through the release of endogenous danger-associated molecular patterns (DAMPs) by ischemic renal cells leads to the initiation of a pro-inflammatory response. Hence, HMGB1, a ubiquitous nuclear protein which is actively released by stimulation of the innate immune system and passively released by ischemic tissues, may trigger TLR4. Waterman *et al*. corroborated the paradigm for MSC immune properties in emphasizing the particular role of TLRs exposure^34^. They observed that TLR3 stimulation of MSC supports their immunosuppressive effects while TLR4 activation provides a pro-inflammatory phenotype, characterized in particular by their dissimilar secretions of cytokines and chemokines^34^. TLR4 priming results in upregulation of pro-inflammatory cytokines, such as IL6 or IL8, while TLR3 priming results in production of anti-inflammatory molecules, such as IL4, IDO, or PGE2. Here, we found we found a significant upregulation of mRNA expression of HMGB1 in MSCD + 1 versus MSCD − 7 ischemic kidneys (Supplementary Figure 1). Furthermore, chemotactic cytokines, including MCP1, are produced by injured renal tubular epithelial cells, subsequently leading to the attraction of inflammatory cells^2^. MCP1 expression was found to be down-regulated in MSCD − 7 group in comparison to control group. Infiltration of kidney parenchyma by inflammatory cells, including MPO-positive neutrophils and F4/80-positive macrophages, is a typical feature of I/R injury^59^. The release of proteases, myeloperoxidases and cytokines by neutrophils, as well as the local production of ROS, further aggravate kidney injury *via* increased vascular permeability and reduced cell integrity^2^. In our study, MSC administration 7 days prior renal I/R was associated with a lower infiltration of neutrophils and macrophages in comparison to saline infusion. Furthermore, macrophage phenotype appears orientated towards M2 immunoregulatory subtype in case of *a priori* exposure to MSC. M2 macrophages are characterized by a low ability to secrete inflammatory cytokines and a high ability to phagocyte apoptotic cells^38^. In line with these observations, IL-6 signaling pathway was found to be down-regulated in MSCD − 7 kidneys compared to control SD − 7 group. The deleterious role of IL-6 in I/R-related AKI has been suggested in murine models: IL-6-knockout mice are less susceptible to I/R AKI, whereas transfer of IL-6-sufficient macrophages by transplantation of wild-type bone marrow into IL-6-deficient mice restore the susceptibility to the ischemic damage^60^.

As a whole, our data suggest that MSC-induced nephro-protective conditioning prior to I/R may involve critical modifications of lipid metabolism, including (i) down-regulated FA biosynthesis, (ii) activated PPARα pathway, (iii) prioritization of FA as source of energy in renal proximal tubule cells, and (iv) decreased availability of free FA, which in turn prevent lipid peroxidation and attenuate renal I/R damage. Additional *in vitro* and *in vivo* studies, including the comparative use of inhibitors of PPARα or CD36, are needed to further decipher the impact of MSC on FA oxidation in renal epithelial cells.

## Materials and Methods

All methods were carried out in accordance with the relevant guidelines and regulations.

### Isolation of MSC from bone marrow

Bone marrow was flushed from both femurs and tibias of male 10-week-old inbred *Lewis* rats (Charles River laboratories). *Lewis* rats are regarded as inbred for several congenic strains. After homogenization in Phosphate-Buffered Saline (PBS, Lonza) +2% fetal bovine serum (FBS, Lonza), the suspension was filtered and centrifuged at 100 g for 10 min. Cells were resuspended in DMEM medium (Lonza) and gently sieved through Ficoll (Healthcare Life Sciences). After an additional centrifugation at 200 g for 45 min at room temperature (RT), mononuclear cells were removed from the gradient interface and suspended in DMEM solution before a new centrifugation of 10 min at 100 g. The cells were then plated in 75 cm^2^ culture flask (Falcon) containing DMEM supplemented with 10% FBS, 1% L-Glutamine (Lonza) and 1% penicillin (Lonza). Cells were then cryopreserved at early (<P3) passages. Following thawing in a water-bath at 37 °C, cells were centrifuged and re-suspended in pre-heated DMEM culture medium. MSC from 3 donors were pooled and expanded together. Several studies have demonstrated that MSC proliferation potential, phenotypic characteristics and ability to differentiate are largely preserved throughout the cryopreservation process^61^. MSC were only used before passage P5.

### Culture and characterization of MSC

MSC were maintained at 37 °C in a humidified 5% CO_2_ incubator. Supplemented DMEM was changed twice a week. When cells reached 80% of confluency, they were split in two 75 cm^2^ culture flasks. On the basis of the conventional criteria^7, 8^, MSC were phenotyped twice, i.e. (i) before their cryopreservation and (ii) before each i.v. injection. Accordingly, the MSC used in the present project adhered to plastic supports and presented a spindle-shaped morphology. MSC were positive for MSC markers as evidenced by flow cytometry, using AlexaFluor-conjugated anti-rat CD29 antibody (BD Pharmingen) and APC-conjugated anti-rat CD90 (BD Pharmingen) antibody. MSC were negative for PE-conjugated anti-CD79a (abcam) antibody, V450-conjugated anti-rat CD45 (BD Horizon) antibody and FITC-conjugated anti-rat CD11b (BD Pharmingen). Cell fluorescence was evaluated by flow cytometry on a FACS Calibur flow cytometer. Data were analysed using FACS Diva softwares. Potential to differentiate into osteoblast, adipocyte, and chondroblast lineages was demonstrated by positive staining for Alizarin Red, Oil Red O and toluidine blue staining, respectively, as previously described^62^. These data concerning MSC quality are summarized in Supplementary Figure 2.

### Rat model of renal ischemia/reperfusion

**Figure 5:**
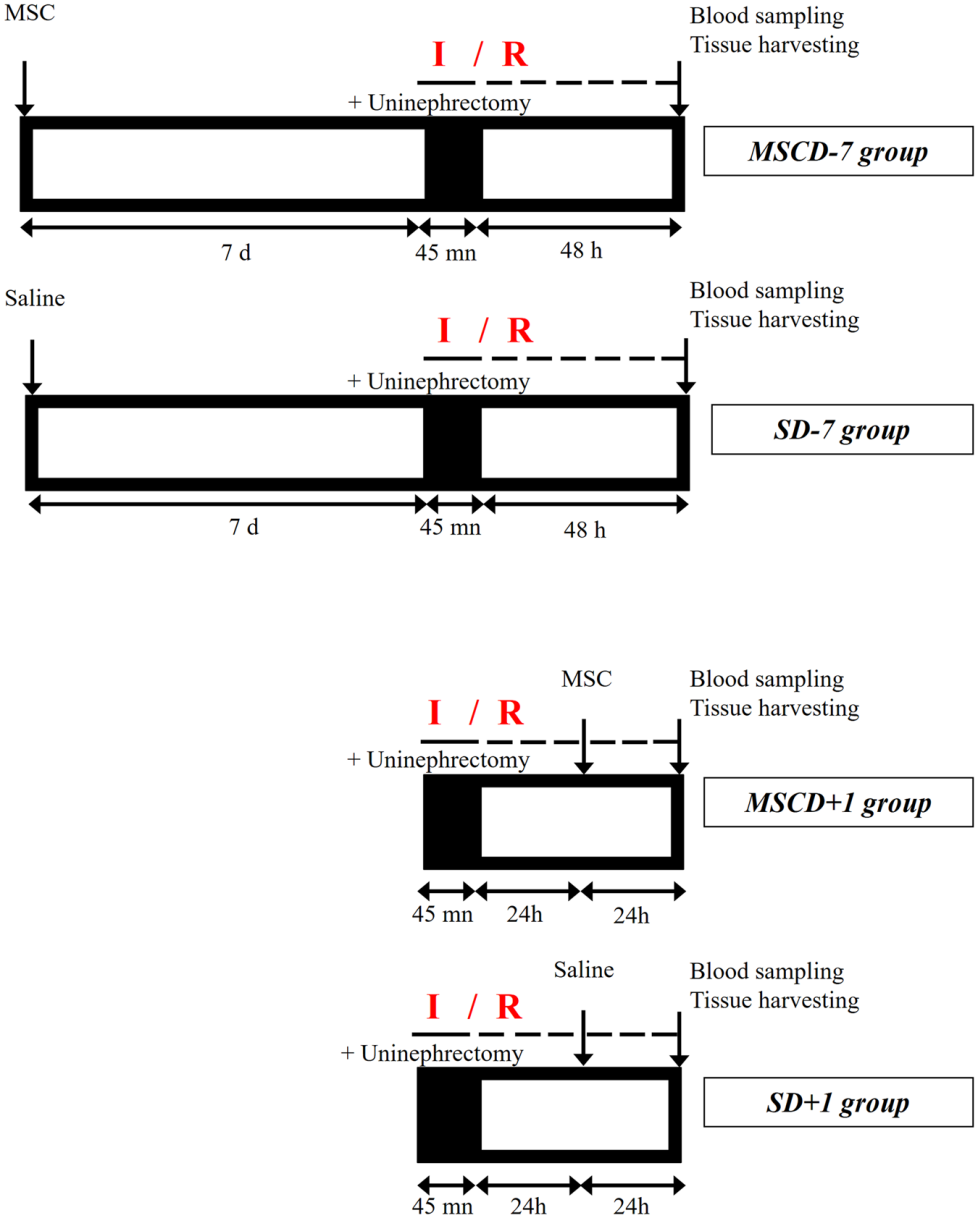
Renal ischemia/reperfusion model. Male *Lewis* rats aged of 8–10 weeks were divided in 4 groups. MSCD − 7 group and MSCD + 1 group received i.v. (tail vein) injection of MSC (1.5 × 10^6^ in 1 ml saline) 7 days before or 1 day after renal ischemia/reperfusion (I/R), respectively. Control groups SD − 7 and SD + 1 received equal volume of saline at similar time-points. Left renal ischemia by clamping the renal pedicle lasted 45 minutes. Right nephrectomy was simultaneously performed. Blood sample and left kidney were collected at 48 h *post* reperfusion.

The Institutional Animal Care and Use Committee of the University of Liege approved the present protocol (#1651). A total of 35 (including 3 dead rats, i.e. 1 in MSCD − 7 group (preoperative) and 2 in MSCD + 1 group (perioperative)) *Lewis* male rats aged of 8–10 weeks were randomly assigned to 4 groups (Fig. 5): MSC injection 7 days before renal I/R (MSCD − 7, n = 11), saline injection 7 days before renal I/R (S D − 7, n = 6), MSC injection 1 day after renal I/R (MSCD + 1, n = 9), saline injection 1 day after renal I/R (S D + 1, n = 6). Rats were anesthetized with pentobarbital (60 mg/kg). Analgesia was performed preoperatively using buprenorphine (0.05 mg/kg). Median laparotomy was performed on heating pads, and a vascular clamp was applied for 45 min on the left renal pedicle. The right kidney was nephrectomized, half-cut and fixed in paraformaldehyde or snap-frozen in liquid nitrogen. During the 45-minute period, the laparotomy area was covered with moistened gauze. Saline (0.5 mL/100 g) was infused intraperitoneally, as well as antibiotics (Enrofloxacin 2.5%, 0.1 mL/rat SC). After surgery, rats were monitored, with *ad libitum* access to food and water. Forty-eight hours after renal I/R, rats were anesthetized. Blood was collected by puncture of the inferior *vena cava*, and centrifuged at 100 g for 5 min at 4 °C. Serum levels of BUN and SCr were measured by enzymatic methods (Roche/Hitachi Cobas). The left kidney was excised, half-cut and fixed in paraformaldehyde or snap-frozen in liquid nitrogen. Snap-frozen (right and left) half-kidneys were grinded into homogeneous powder using B.Braun Mikro-Dismembrator before protein or mRNA extractions for further analyses.

### Administration of MSC

MSC were detached from culture flasks by Trypsin-EDTA, and centrifuged at 200 g for 5 min in DMEM. Cells were counted in a Thoma chamber, and 1.5 × 10^6^ cells were suspended in 1 mL of sterile saline (in order to avoid infusing rats with culture medium). The dose of 1.5 × 10^6^ cells *per* rat was chosen on the basis of previous preclinical investigations^4^. Cell suspensions were slowly injected into the tail vein 7 days before (MSCD − 7) or 1 day after (MSCD + 1) renal I/R (Fig. 5). MSC were i.v. injected in parallel in all groups over a 2-day period. Quality of MSC administered in MSCD − 7 and MSCD + 1 groups can thus be technically regarded as identical. Control rats were infused with an equal volume of saline at the same time-points.

### Histology and immunostaining

Sections were dewaxed and gradually hydrated before hematoxylin-eosin (HE) and Periodic Acid Schiff (PAS) staining. I/R-induced acute tubular necrosis was blindly scored by a renal pathologist following Jablonski score^63^. For immunohistochemistry (IHC), sections were subjected to antigen retrieval in sodium citrate buffer (pH 6.0, Dako #S2031) or Target buffer (Dako #S1699) or EDTA buffer (Dako #S2367). Endogenous peroxidase activity was blocked with 3% hydrogen peroxide (Merck 30%, #107209) for 20 min at RT. Non-specific binding was constrained by incubation for 30 min with either normal goat serum or for 10 min with protein block reagent (Dako #X0909). Then, sections were incubated for 60 min at RT with primary antibodies: monoclonal mouse anti-PCNA (Dako, #M0879; sodium citrate buffer, NGS, primary antibody 1/150 for one hour at room temperature (RT)); anti-HSP70 (Enzo LifeScience, 810F; sodium citrate buffer, protein block reagent, primary antibody 1/50 for one hour at RT); anti-MDA (abcam #ab6463; Target buffer, NGS, primary antibody 1/1000 for one hour at RT); anti-Myeloperoxidase (abcam #ab9535; Target buffer, NGS, primary antibody 1/200 overnight); anti-CD36 (abcam #ab133625; sodium citrate buffer, protein block reagent, primary antibody 1/100 for one hour at RT); F4/80 (abcam #74383; EDTA buffer, protein block reagent, primary antibody 1/1000 for one hour at RT); CD163 (abcam #186422; sodium citrate buffer, protein block reagent, primary antibody 1/500 for one hour at RT). After washing, sections were incubated for 30 min with goat anti mouse or rabbit biotin-conjugated secondary antibody (1/400), washed and exposed to horseradish peroxidase-conjugated streptavidin (1/500) for 30 min. Immunoreactivity was detected using DAB (Dako #K3468) or AEC (Dako #K3464). Apoptosis was studied using ApopTag Plus Kit (Millipore #S7101) following the manufacturer’s instructions. IHC scoring was achieved blindly in duplicate on digital images (NanoZoomer 2.0 HT, Hamamatsu^®^): ten randomly selected fields of the cortico-medullary region (original magnification, ×400) were considered per kidney.

### Immunoblotting

Half-kidneys were disrupted and homogenized by oscillations (Mikro-dismembrator S, B. Braun Biotek International) for 1 min. Protein extraction was performed using ice-cold TEN-T buffer including protease and phosphatase inhibitors (Roche). TEN-T buffer includes: NaCl [5 M], EDTA [0.5 M], Tris-HCl [1 M], 1% Triton X-100. Supernatant was collected after centrifugation at 200 g for 30 min at 4 °C. Protein concentration was determined using Bradford method. Protein lysates were mixed with Laemmli buffer (1:4) and heated for 2 min at 95 °C. Samples were loaded and separated at 100 V on stain-free SDS gel electrophoresis gels (Bio-Rad) (30 μg/lane). Gels were exposed to UV light for 5 min (ChemiDoc MP system, Bio-Rad). Proteins were transferred to PVDF membranes using the Trans-Blot Turbo Transfer System for 7 min at RT. Blots were blocked with 5% milk in Tris-buffered saline with Tween 20 (TBS-T) for 1 hour, and incubated overnight at 4 °C with primary antibodies: PPARα (abcam ab8934, 1/1000), p-PPAR alpha, MDA (abcam ab6463, 1/1000), CD36 (abcam ab133625, 1/100), 4-HNE (abcam 46545, 1/500). Blots were rinsing five times with TBS-T for 5 min, and incubated with appropriate HRP-conjugated anti-rabbit or anti-mouse secondary antibodies (1/4000) for 90 min at RT. After rinsing, chemiluminescent signals were captured by ChemiDoc MP System after applying chemiluminescent substrate (SuperSignal West Femto Maximum Sensitivity Substrate, Thermoscientific) on blots. Immunoreactive signals were quantified using Bio-Rad^®^ stain-free technology after normalization to total protein content^64^, as described in Supplementary Figure 3. The use of gels/blots in the figures complies with the digital image and integrity policies (www.nature.com/srep/policies/index.html#digital-image).

### RNA sequencing and real-time quantitative polymerase chain reaction (RT-qPCR)

Messenger RNAs were extracted from the right kidneys of rats exposed (MSCD − 7) or not-exposed (SD − 7) to MSC 7 days before sampling. These kidneys did not suffer from I/R, and were harvested before the 45-min ischemia of the contralateral kidneys. After homogenization in 1 mL Tripure solution (Roche) and 200 μL of chloroform, lysates were centrifuged at 200 g at 4 °C for 15 min. The upper aqueous phase was diluted with 500 μL of isopropyl alcohol. The mixtures were centrifuged at 200 g at 4 °C for 10 min, and pellets were suspended in 500 μL of 70% ethyl-alcohol before centrifugation at 100 g at 4 °C for 5 min. Pellets were finally dissolved in 100 μL RNase-free water. RNA concentration and purity were assessed using NanoDrop Lite spectrophotometer (Thermo Scientific). All RNA samples had an absorbance [260 nm/280 nm] ratio above 1.8. Libraries were prepared for each sample using Truseq mRNA stranded kit from Illumina and sequenced on a NextSeq. 500 sequencer producing an average of 20 million 2 × 75-bp reads *per* library. BaseSpace Sequence Hub Illumina was used for the evaluation of the sequencing data. Reads were mapped onto the rat reference genome (rn5) using TopHat. Quality control metrics meet the expectations for this type of libraries, with especially (i) a percentage of reads that align to the selected reference genome >93% for each sample, (ii) a median coefficient of variation of coverage of the 1000 most highly expressed transcripts lower than 0.4 for each sample (as a measure of the uniformity of coverage across transcripts) and (iii) a percentage of reads that align to the correct strand (compared to reference genome annotation) higher than 99.4% for each sample (Supplementary Table 1). Library quality were also confirmed by Picard analysis showing the expected coverage for mRNA transcripts (Supplementary Figure 3a) and the absence of degradation (Supplementary Figure 3b). After running TopHat, resulting data were transferred to Cufflinks and Cuffmerge to generate a transcriptome assembly. Identification of genes differentially regulated was then performed with Cuffdiff^26^. BaseSpace Sequence Hub Illumina was used for the evaluation of the sequencing data. To interpret the gene lists derived from RNA sequencing, functional enrichment analysis was performed using Database for Annotation, Visualization and Integrated Discovery (DAVID)^27^ and WEB-based GEne SeT AnaLysis Toolkit (WebGestalt)^28^. Significantly differentially expressed genes were classified into gene ontology categories with WebGestalt (2015, October). Relevant pathways were further detected using an Over Representation Analysis with WebGestalt, based on the proportion of differential expressed genes within a given pathway surpassing the proportion of genes that could be randomly expected (WikiPathways as enrichment category; GeneSymbol as ID type)^65^. Afterwards, cDNAs were generated using Reverse Transcription Kit (Promega) according to manufacturer’s instruction. Primers used for RT-qPCR are listed in Table 2. Semi-quantitative mRNA expression levels were calculated using threshold cycle (Ct) values following the classical 2^−Δ*C*T^ equation. The housekeeping gene used for RT-qPCR was GAPDH.

**Table 2:** Primers used for qPCR.

Gene	Direction	Primer sequences	Size of PCR product	GenBank accession number
*Bax*	Forward	GCTGACATGTTTGCAGACGG	865 bp	NM_017059.2
	Reverse	GTGTCCAGCCCATGATGGTT		
*Bcl-2*	Forwad	CCGGGAGAACAGGGTATGATAA	1179 bp	NM_016993.1
	Reverse	CCCACTCGTAGCCCCTCTG		
*Casp3*	Forward	GGAGCTTGGAACGCGAAGAA	2484 bp	NM_012922.2
	Reverse	CGACATCGGTACCATTGCGA		
*Hsp70*	Forward	TCAGCGAGGCTGACAAGAAG	5918 bp	NM_212504.1
	Reverse	GCAGCCATCAAGAGTCTGTCT		
*Icam1*	Forward	CGGTGCTCAGGTATCCATCC	2602 bp	NM_012967.1
	Reverse	CTCGCTCTGGGAACGAATACA		
*IL-6*	Forward	TTGCCTTCTTGGGACTGATGT	1045 bp	NM_012589.2
	Reverse	TACTGGTCTGTTGTGGGTGGT		
*Kim-1*	Forward	CGCAGAGAAACCCGACTAAG	3150 bp	XM_008767666.2
	Reverse	CAAAGCTCAGAGAGCCCATC		
*Mcp-1*	Forward	GCTGTAGTATTTGTCACCAAGCTC	155 bp	NM_031530.1
	Reverse	GGTGCTGAAGTCCTTAGGGT		
*Tnf α*	Forward	ATGGGCTCCCTCTCATCAGT	1724 bp	XM_008772775.2
	Reverse	GCTTGGTGGTTTGCTACGAC		
*Acaca*	Forward	ATTGGGGCTTACCTTGTCCG	7038 bp	NM_022193.1
	Reverse	ACTGTGCACGTTCTTAGGCA		
*Acly*	Forward	GCCAGGGAGCTGGGTTTAAT	4331 bp	NM_016987.2
	Reverse	TGCCCATGATCAGGTTCCCC		
*Fasn*	Forward	TTCAGGGAACGGGTATTGCC	9136 bp	NM_017332.1
	Reverse	AATGTCACGCCTTGCTCCTT		
*Gab1*	Forward	CCGAACCGATTCAGGAACCA	4177 bp	NM_001108444.1
	Reverse	ACCTAGAGGAGTCCCGAGC		
*Hmgb1*	Forward	TTGAGCTCCATAGAGACAGCG	433 bp	NM_001109373.1
	Reverse	GCCTTTGATTTTTGGGCGGT		
*iNos*	Forward	CTAGTCAACTACAAGCCCCACG	291 pb	NM_012611.3
	Reverse	TCGATGGAGTCACATGCAGC		
*Arg*	Forward	AACACTCCCCTGACAACCAG	274 pb	NM_017134.3
	Reverse	CCAGCAGGTAGCTGAAGGTC		
*Gapdh*	Forward	ATCCCGCTAACATCAAATGG	170 pb	NM_017008.4
	Reverse	GTGGTTCACACCCATCACAA		

### Statistical analyses

Data were expressed as mean ± standard deviation (SD). One-way analysis of variance was performed using MedCalc^®^ (MedCalc, software, Mariakerke, Belgium). Multiple 2-to-2 comparisons were performed using Dunn- Šídák test. Chi-square and Mann-Whitney U tests were used to compare discrete variables. A *p* value ≤ 0.05 was considered as statistically significant.

## Electronic supplementary material

Supplementary Information
