# Supplementary material for: Administration of mesenchymal stromal cells before renal ischemia/reperfusion attenuates kidney injury and may modulate renal lipid metabolism in rats
Source: Sci Rep. 2017 Aug 17;7:8687. doi: 10.1038/s41598-017-08726-z (PMC5561049; doi:10.1038/s41598-017-08726-z)
Supplement: Supplementary file 1 — Supplementary Information [file 41598_2017_8726_MOESM1_ESM.doc]

**Administration of mesenchymal stromal cells before renal ischemia/reperfusion attenuates kidney injury and may modulate renal lipid metabolism in rats**

Erpicum Pauline (1, 2), Rowart Pascal (1), Poma Laurence (1), Krzesinski Jean-Marie (1, 2), Detry Olivier (1, 3), and Jouret François (1, 2,*)

1. Groupe Interdisciplinaire de Génoprotéomique Appliquée (GIGA), Cardiovascular Sciences, University of Liège, Liège, Belgium
2. Division of Nephrology, University of Liège Hospital (ULg CHU), Liège, Belgium
3. Department of Abdominal Surgery and Transplantation, University of Liège Hospital (ULg CHU), Liège, Belgium

*** Corresponding author:**

François JOURET, MD, PhD

University of Liège Academic Hospital (ULg CHU), Division of Nephrology

Avenue Hippocrate, 13 – B4000 Liège, Belgium

Tel: +32.4.366.25.40; Fax: +32.4.366.21.37

E-mail: [francois.jouret@chu.ulg.ac.be](mailto:francois.jouret@chu.ulg.ac.be)

**Supplementary Figure S1.**

**(a)** Serum creatinine levels in MSCD-7 and MSCD+1 groups. **(b)** Blood urea nitrogen (BUN) levels in MSCD-7 and MSCD+1 groups. **(c)** Immunohistochemistry and quantification for Apoptag-, PCNA-, HSP70-, MPO-positive, F4/80- and CD163-positive cells in MSCD-7 and MSCD+1 kidneys at the cortico-medullary junction. **(d)** Effect of MSC administration on renal mRNA expression levels of *Bax, Bcl-2, Casp3, Hsp70, Kim-1, Icam-1, Tnf alpha, Il-6* and *Hmgb1* after 45-min ischemia followed by 48-hour reperfusion in MSCD-7 and MSCD+1 groups. Data are presented as mean ± standard deviation. Significant differences are indicated, * *p≤*0.05, ** *p≤*0.01 and *** *p≤*0.001.


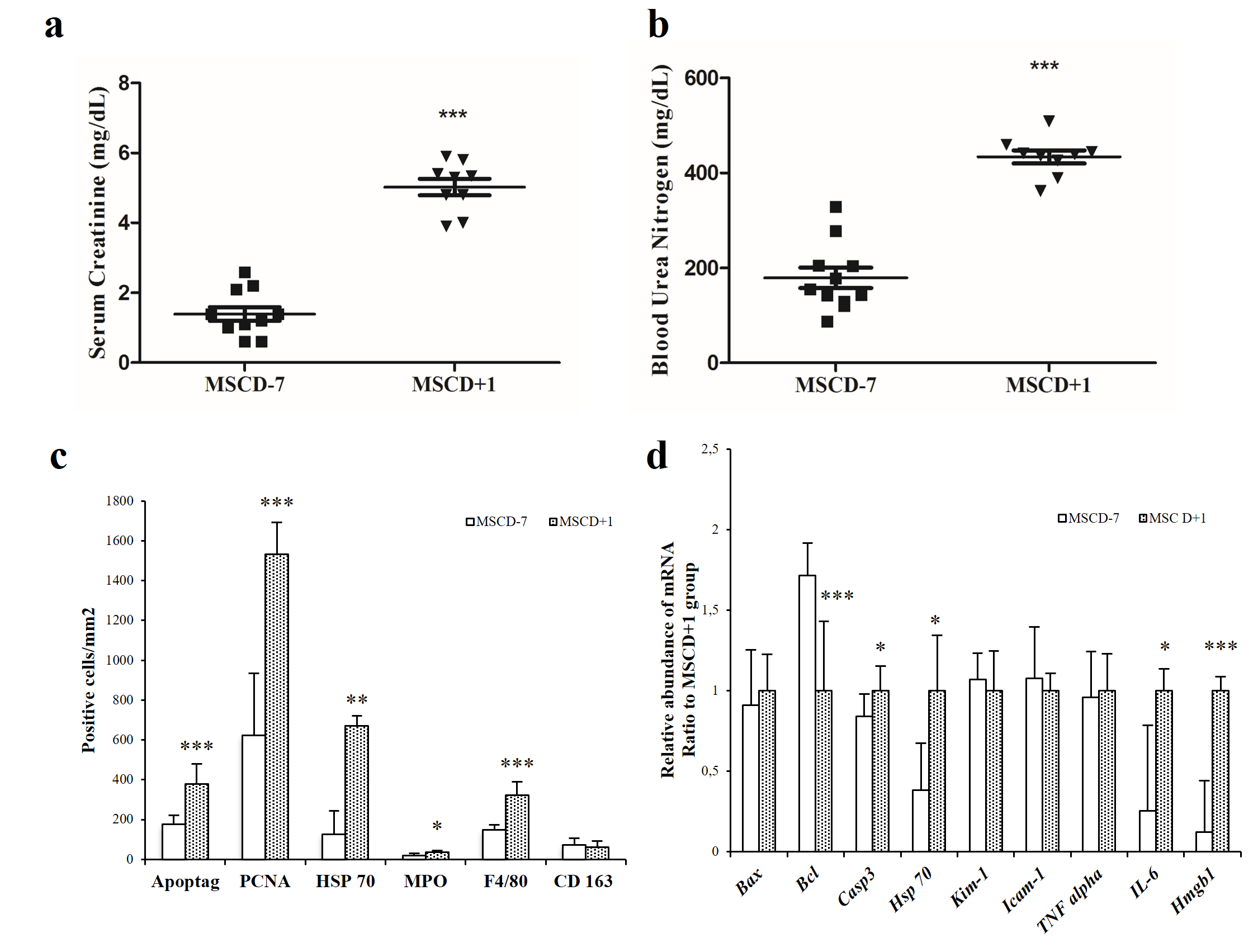


**Supplementary Figure S2.** Quality criteria of mesenchymal stromal cells (MSC)

**(a)** MSC adhere to plastic support, as a heterogeneous cell population with spindle-shaped fibroblastic morphology: (a1) MSC at low cellular density; (a2) MSC at full confluency **(b-c)** Flow cytometry is performed on a FACS using AlexaFluor-conjugated anti-rat CD29 antibody, APC-conjugated anti-rat CD90 antibody, V450-conjugated anti-rat CD45 antibody, FITC-conjugated anti-rat CD11b and PE-conjugated anti-CD79a antibody: (b) MSC are positive for CD90, but negative for CD79, CD11b and CD45; (c) MSC are positive for CD29, but negative for CD79, CD11b and CD45. **(d)** MSC differentiation into adipocytes (Oil-Red), osteoblasts (Alizarin Red) and chondroblasts (Toluidine blue) following exposure to appropriate induction media.


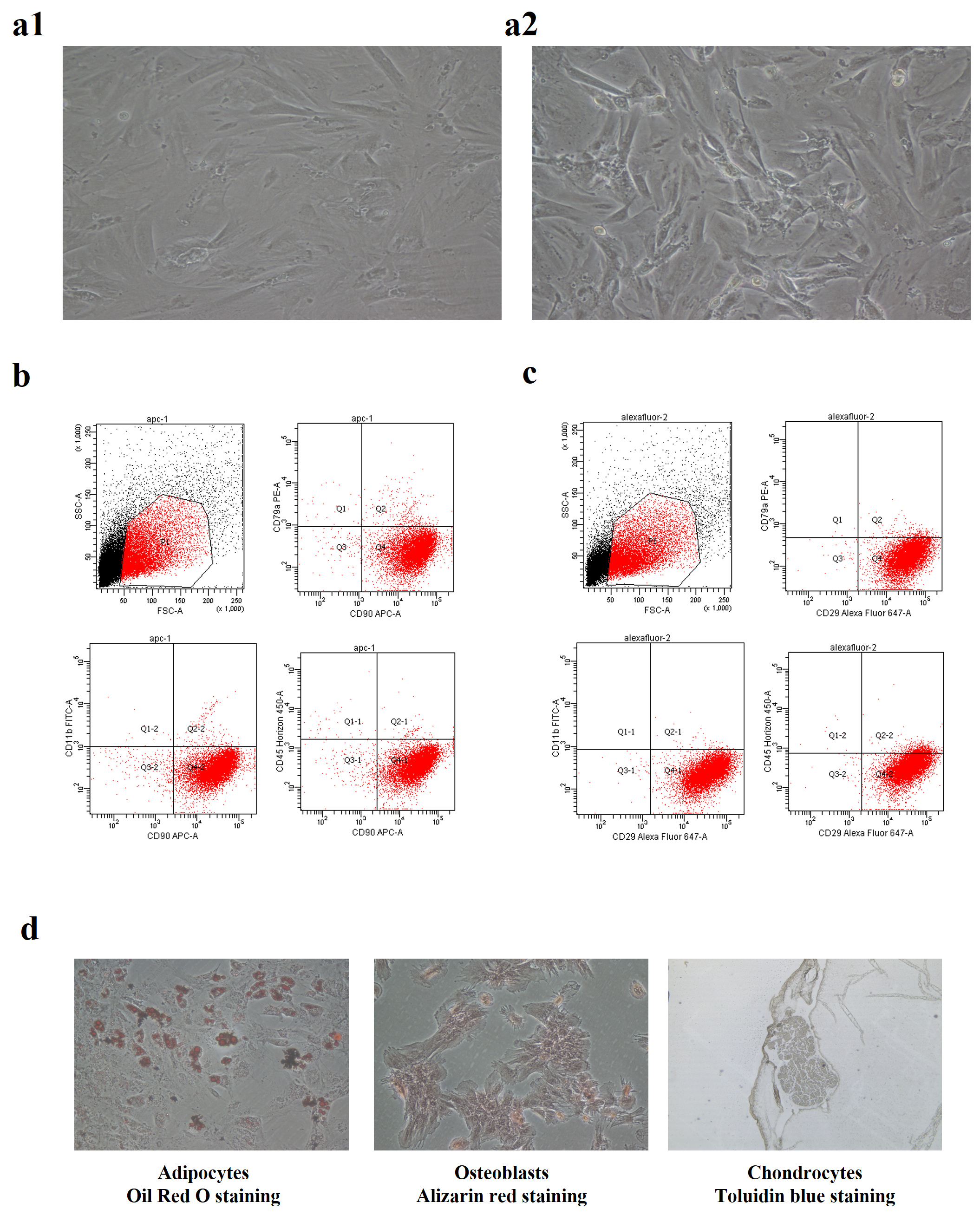


**Supplementary Figure S3.** **(a)** Metrics specific to individual samples within the run during RNA-sequencing analyzed with Picard tools <https://broadinstitute.github.io/picard/>. TopHat Alignment distribution Graph illustrating the percentage of sequences aligned to the respective genomic regions. Lines 5-D568 to 5-D573 refer to MSCD-7 samples, whereas lines 5-D574 to 5-D579 refer to SD-7 samples. **(b)** TopHat Transcript coverage showing the average relative coverage for each library at each relative position along transcript length. **(c)** Immunoblotting quantification using stain-Free Technology after normalization to total protein content. From left to right: gel at the end of protein migration, exposed to UV light; protein onto the PVDF membrane; immunoblotting for PPAR alpha; and quantification of the chemiluminescent signal using ChemiDoc MP System (Bio-Rad®) and Image Lab 4.1 software. The immunoreactive signal in each lane is normalized to the corresponding total protein amount detected in the stain-free image of the PVDF membrane.


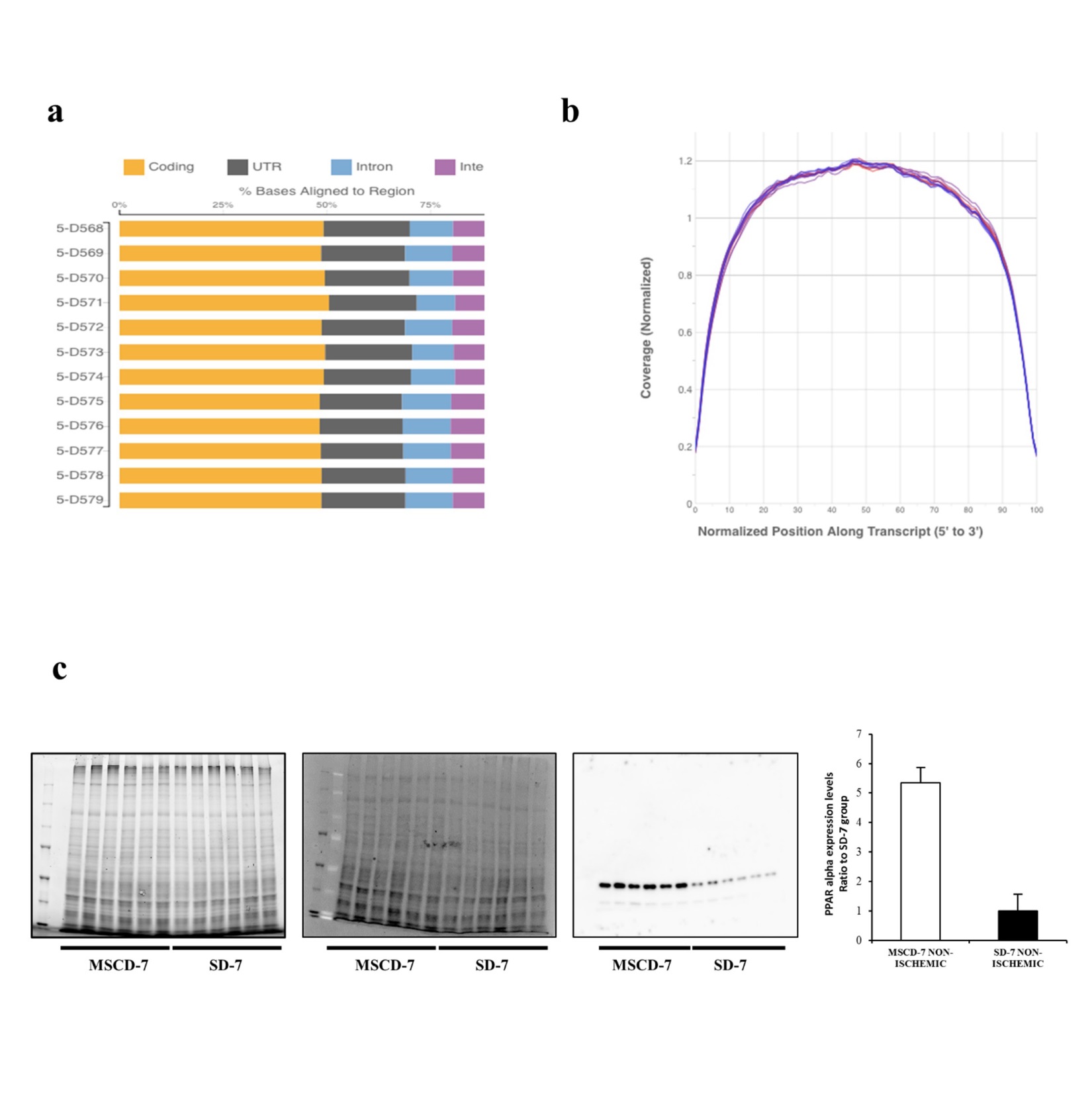


**Supplementary Table 1. Overview of the alignment statistics for all samples provided by the TopHat App.**

Lines 5-D568 to 5-D573 refer to MSCD-7 samples, whereas lines 5-D574 to 5-D579-D refer to SD-7 samples. Reads, number and length of reads; Number of Reads, total number of reads passing filter for this sample; % Total Aligned, percentage of reads passing filter that aligned to the reference, including abundant reads; % Abundant, percentage of reads that align to abundant transcripts, such as mitochondrial and ribosomal sequences; % Unaligned, percentage of passed filter reads that do not align to the reference; Median CV Coverage Uniformity, the median coefficient of variation of coverage of the 1000 most highly expressed transcripts, as reported by the CollectRnaSeqMetrics utility from Picard tools; % Stranded, percentage of reads that align to the correct strand, as reported by the CollectRnaSeqMetrics utility from Picard tool.

<https://www.illumina.com/content/dam/illuminamarketing/documents/products/other/user-guide-rna-seq.pdf>

|  | **Reads** | **Numbers of Reads** | **% Total aligned** | **% Abundant** | **% Unaligned** | **Median CV Coverage Uniformity** | **% Stranded** |
| --- | --- | --- | --- | --- | --- | --- | --- |
| 5-D568 | 76/76 | | 18,649,390 |  | | --- | --- | | 93.83% | 15.69% | 6.17% | 0.40 | 99.47% |
| 5-D569 | 76/76 | 17,822,954 | 93.35% | 15.64% | 6.65% | 0.39 | 99.50% |
| 5-D570 | 76/76 | 18,315,647 | 93.59% | 14.13% | 6.41% | 0.39 | 99.50% |
| 5-D571 | 76/76 | 18,706,219 | 93.71% | 15.31% | 6.29% | 0.39 | 99.52% |
| 5-D572 | 76/76 | 18,710,587 | 93.60% | 14.84% | 6.40% | 0.40 | 99.48% |
| 5-D573 | 76/76 | 18,989,820 | 93.56% | 15.99% | 6.44% | 0.39 | 99.50% |
| 5-D574 | 76/76 | 18,478,314 | 93.67% | 15.71% | 6.33% | 0.39 | 99.47% |
| 5-D575 | 76/76 | 18,808,368 | 93.80% | 14.31% | 6.20% | 0.40 | 99.44% |
| 5-D576 | 76/76 | 22,091,476 | 94.33% | 14.23% | 5.67% | 0.39 | 99.46% |
| 5-D577 | 76/76 | 22,603,992 | 94.18% | 14.67% | 5.82% | 0.39 | 99.45% |
| 5-D578 | 76/76 | 20,578,605 | 93.69% | 14.37% | 6.31% | 0.39 | 99.47% |
| 5-D579 | 76/76 | 19,092,859 | 93.51% | 15.00% | 6.49% | 0.39 | 99.49% |
